# Case Report: An MDT-guided multimodal protocol for large-area stroke plateau — integrating neuromodulation with multimodal assessment

**DOI:** 10.3389/fresc.2026.1803123

**Published:** 2026-10-09

**Authors:** Xiao-li Wu, Xiao-qian Xia, Chang-qing Ye, Yun-lei Wang, Xing-zhu Li, Li-xu Liu, Fu-biao Hang, Jing-song Liu, Yi-chuan Song, Yue Zhang, Xiao-xia Du

**Affiliations:** 1 Department of Neurology Rehabilitation, Beijing Bo'ai Hospital, China Rehabilitation Research Center, Beijing, China; 2 School of Rehabilitation Medicine, Capital Medical University, Beijing, China; 3 Department of Occupational Therapy, Beijing Bo'ai Hospital, China Rehabilitation Research Center, Beijing, China; 4 Institute of Rehabilitation Engineering, Beijing Bo'ai Hospital, China Rehabilitation Research Center, Beijing, China; 5 Music Therapy Center, Beijing Bo'ai Hospital, China Rehabilitation Research Center, Beijing, China; 6 Department of Rehabilitation Assessment, Beijing Bo'ai Hospital, China Rehabilitation Research Center, Beijing, China

**Keywords:** stroke, transcranial magnetic stimulation, functional near-infrared spectroscopy, electroencephalography, power spectral density, rehabilitation

## Abstract

**Objective:**

This case report investigates the feasibility and potential value of an individualized precision rehabilitation protocol for large-area stroke, integrating multimodal neuromodulation under the guidance of a multidisciplinary team (MDT) and dynamic assessment.

**Methods:**

A 38-year-old male with right hemiplegia and global aphasia following large left hemispheric infarction underwent an 8-month MDT-managed program. A closed-loop “assess-decide-intervene-re-assess” framework was implemented, featuring periodic multimodal evaluations (clinical scales, DTI, fMRI, EEG, 3D gait analysis). The intervention dynamically combined conventional therapy with staged neuromodulation: repetitive Transcranial Magnetic Stimulation (rTMS) for aphasia and intermittent Theta Burst Stimulation (iTBS) for motor disorder, Brain-Computer Interface (BCI)-robotic training and task-oriented Virtual Reality (VR) for upper limb function, and biomechanical correction with custom insoles based on gait analysis.

**Results:**

Notable functional improvements were observed: LOTCA increased by 16 points, CRRCAE subscale improved by 60%–90%, FMA increased from 20 to 56, FMA-B reached 12, and MBI improved from 40 to 91. Neuroimaging revealed increased fractional anisotropy (FA) in the corpus callosum and right corticospinal tract, and elevated ALFF/ReHo in right frontal-temporal areas. Event-related desynchronization (ERD) decreased during BCI training, and gait parameters improved.

**Conclusion:**

In this patient with large-area stroke who showed limited response to conventional rehabilitation, an MDT-guided, dynamically adjusted multimodal intervention was temporally associated with concurrent recovery across cognitive, language, motor, and daily living domains. The concomitant neural changes suggest possible trans-hemispheric network reorganization. These observations are hypothesis-generating and, while limited by the single-case design, provide a preliminary rationale for future investigations into precision neurorehabilitation approaches in similar complex cases.

**Clinical trial registration:**

ChiCTR2300069203.

## 1 Introduction

Large-area stroke resulting in the severe triad of hemiplegia, global aphasia, and cognitive impairment represents a formidable challenge in neurorehabilitation, leading to profound disability (1)^.^ Conventional therapies often plateau, limited by an inability to dynamically adapt to individual neuroplasticity trajectories (2).

Recent scholarship has underscored the imperative for a paradigm shift in neurorehabilitation. An editorial in The Lancet Neurology emphasized that complex diseases must be addressed through integrated, multidisciplinary approaches, and that neurorehabilitation should serve as a cornerstone of recovery rather than an afterthought (3). Del Felice et al. further articulated that neurorehabilitation is a forerunner in the shift towards systems medicine, advocating for personalized interventions that recognize that identical training protocols may yield vastly different outcomes across individuals, and calling for the integration of biological and environmental factors within the ICF framework—moving beyond treating the “lesion” to driving biological repair through activity and participation, thereby achieving true bio-psycho-social integration (4). The multidisciplinary team (MDT) model has been increasingly recognized as essential for managing the complexity of post-stroke impairments, with evidence supporting its benefits in functional outcomes, psychological well-being, and care coordination (5–7). However, most MDT models still operate on fixed protocols or periodic reviews without integrating real-time, biomarker-guided neuromodulation within a responsive, closed-loop framework.

While the integration of advanced neuromodulation techniques [e.g., Transcranial Magnetic Stimulation(rTMS), Brain-Computer Interface(BCI), Virtual Reality(VR)]shows promise for engaging residual networks,and the multidisciplinary team (MDT) model is crucial for managing complexity, a critical translational gap remains (2, 4). There is a lack of integrated frameworks that synergize MDT coordination with quantitative, biomarker-guided neuromodulation within a responsive, closed-loop system capable of personalizing therapy to the patient's evolving neural state (8).

This case report represents an attempt to operationalize the Lancet Neurology vision—implementing an MDT-led, dynamically adjusted, multimodal neuromodulation protocol informed by serial quantitative assessments within a closed-loop “assess-decide-intervene-reassess” framework. We illustrate the application of this paradigm in a young adult with large left hemispheric stroke, highlighting its potential as a hypothesis-generating framework for rehabilitating similar complex cases.

## 2 Case description and methods

The patient was enrolled in a larger registered cohort study (ChiCTR2300069203); this report provides a detailed granular analysis of one representative individual from that cohort to illustrate the protocol's application. Ethical approval was obtained from the Institutional Review Board of China Rehabilitation Research Center (Approval No: 2022-140-01). Written informed consent was provided by the patient's legal guardian.

### 2.1 Patient presentation

A 38-year-old, previously healthy, right-handed male was admitted to our neurorehabilitation unit one month after sustaining a large left fronto-temporo-parietal infarction,confirmed by magnetic resonance imaging (see Supplementary Figure S1), secondary to spontaneous internal carotid artery dissection. According to the transfer records from the community hospital, no significant functional gains were documented during the three weeks of conventional rehabilitation prior to admission. The admission assessment, structured using the International Classification of Functioning, Disability and Health (ICF) framework (Figure 1), revealed the classic and severe triad of deficits associated with large dominant hemisphere stroke. Neurological examination confirmed global aphasia, with communication limited to stereotypical phonemes and an inability to perform any formal language task (comprehension, repetition, naming, reading, writing). Formal cognitive evaluation was precluded by the severe aphasia; therefore, the baseline LOTCA assessment (reported in Table 1) included only non-verbal visual-spatial and motor organization subtasks that the patient could complete with non-verbal responses. This methodological constraint is acknowledged in the limitations section.

**Figure 1:**
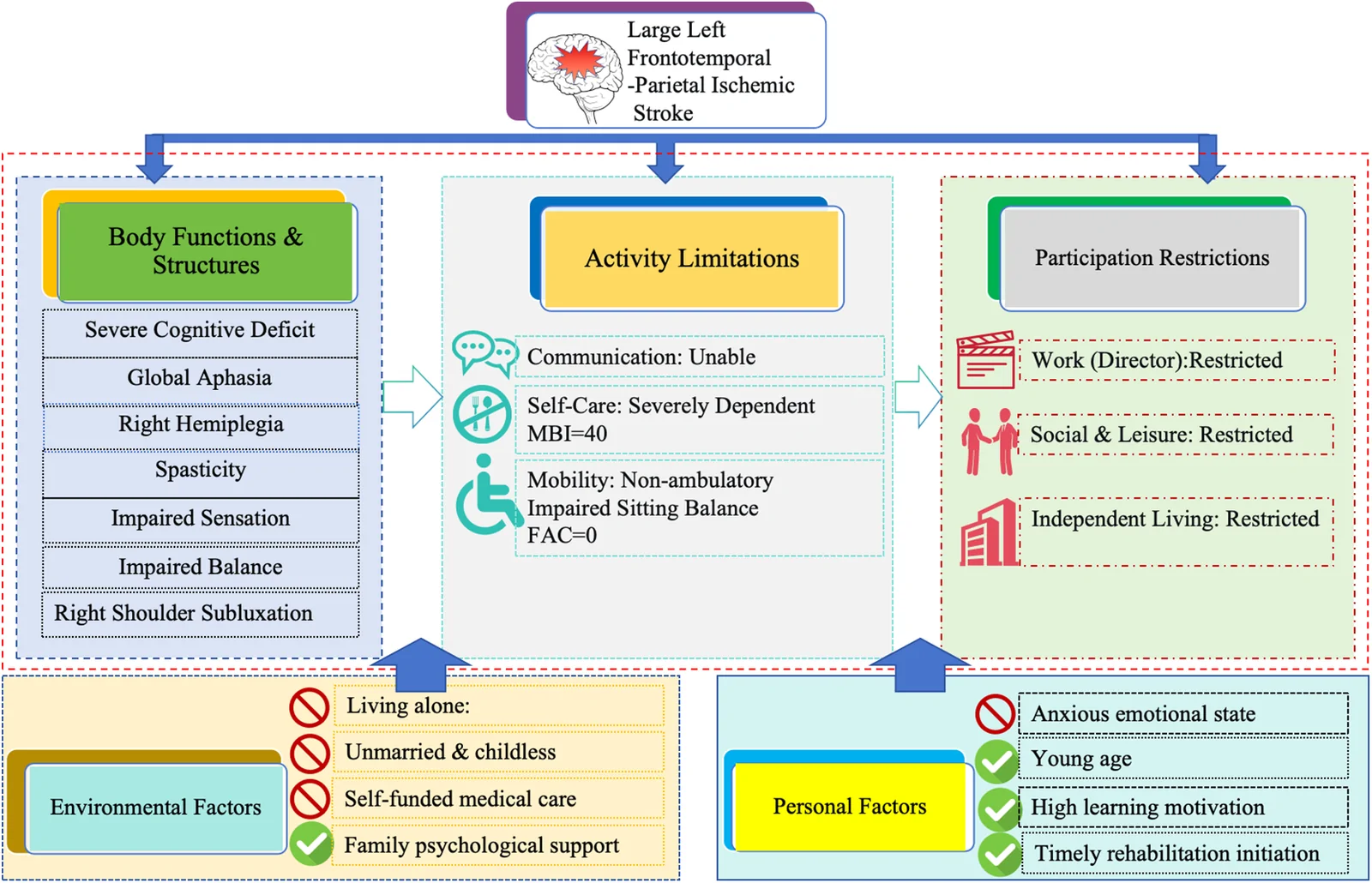
Comprehensive functional profile at admission, conceptualized within the international classification of functioning, disability and health (ICF) framework. This schematic synthesizes the patient's multi-dimensional deficits following a large left hemispheric stroke. Body Functions & Structures: The central illustration highlights the primary CNS lesion (left fronto-temporo-parietal infarction) and the resulting impairments: global aphasia, severe cognitive deficit, right hemiplegia with spasticity, sensory loss, impaired balance, and the structural complication of right shoulder subluxation. The Activity Limitations component quantifies the resultant severe disabilities: complete dependence in communication, severely limited self-care (MBI=40), non-ambulatory status (FAC=0), and impaired sitting balance. The Participation Restrictions component illustrates the consequent inability to engage in life roles, including work (as a director), social interactions, and independent living. This holistic profile underscores the necessity for a comprehensive, multi-targeted rehabilitation strategy. ICF, International Classification of Functioning, Disability and Health; MBI, modified Barthel index; MAS, modified Ashworth scale; FAC, functional ambulation category. Activity Limitations: Icon-based representation shows severe dependence in Communication (crossed speech bubble), Self-Care (crossed dressing icon; MBI=40), and Mobility (wheelchair icon; non-ambulatory). Participation Restrictions: Icons indicate major restrictions in Work (director's chair with lock), Social & Leisure Activities (broken link), and Independent Living (locked house). This visualization underscores the holistic impact of the stroke and the rationale for a comprehensive MDT approach. ICF, International Classification of Functioning, Disability and Health; MBI, modified Barthel index; MAS, modified Ashworth scale.

**Table 1:** Alterations in comprehensive functional assessments throughout the rehabilitation therapy period.

Assessment items	At admission	After 4 weeks of treatment	After 12 weeks of treatment	After 16 weeks of treatment	After 20 weeks of treatment	At discharge	4 weeks after discharge
MMSE	NCC	NCC	7	13	16	20	20
NIHSS	11	9	8	5	4	4	4
LOTCA	46	NC	53	NC	NC	NC	62
FMA	20	34	39	40	42	53	54
FMA-B	7	10	11	11	11	11	12
Brunnstrom (Upper Limb)	Ⅱ	Ⅲ	Ⅲ	Ⅲ	Ⅳ	Ⅳ	Ⅳ
Brunnstrom (Hand)	Ⅱ	Ⅲ	Ⅲ	Ⅲ	Ⅲ	Ⅲ	Ⅲ
Brunnstrom (Lower Limb)	Ⅲ	Ⅲ	Ⅲ	Ⅳ	Ⅳ	Ⅳ	Ⅳ
HAMA	NC	NC	10	7	6	4	2
HAMD	NC	NC	8	6	6	1	1
HFAC	0	1	1	2	3	3	4
MBI	40	50	58	86	86	91	91
MAS(Upper Limb)	Ⅰ+	Ⅱ	Ⅱ	Ⅰ+	Ⅰ+	Ⅰ+	Ⅰ+
MAS(Hand)	Ⅰ+	Ⅱ	Ⅱ	Ⅰ+	Ⅰ+	Ⅰ+	Ⅰ+
MAS(Lower Limb)	Ⅰ	Ⅰ+	Ⅰ+	Ⅰ	Ⅰ	Ⅰ	Ⅰ

NCC, non-compliant collection; NC, not checked; MMSE, mini-mental status examination; NIHSS, National Institutes of Health Stroke Scale; LOTCA, Loewenstein occupational therapy cognitive assessment; FMA, Fugl–Meyer assessment; FMA-B, Fugl–Meyer assessment of balance scale; HAMA, Hamilton anxiety scale; HAMD, Hamilton depression scale; HFAC, Holden functional ambulation classification; MBI, modified Barthel index; MAS, modified Ashworth scale.

Motor examination identified right-sided hemiplegia (Brunnstrom stages: upper limb III, hand II, lower limb III), with spasticity in the right upper limb flexors [Modified Ashworth Scale (MAS) grade 1+], a right glenohumeral subluxation (one fingerbreadth), globally hyperactive tendon reflexes, and positive pathological signs (right-sided ankle clonus, Hoffmann and Babinski signs). Sensory testing suggested impairment but was limited by cooperation. Balance was impaired, with independent sitting balance graded as level 1.

Consequently, his functional status was severely compromised, characterized by severe dependence in all basic and instrumental activities of daily living [Modified Barthel Index (MBI) score: 40/100], non-ambulatory status, and an inability to engage in his prior work or social roles. As a professional film director, his deficits rendered him unable to perform any aspect of his work—a role requiring high-level communication, spatial reasoning, executive planning, and social interaction.

### 2.2 MDT framework and dynamic assessment protocol

A structured, closed-loop rehabilitation protocol was implemented, as visualized in Figure 2. A dedicated multidisciplinary team conducted comprehensive multimodal evaluations at scheduled four-week intervals. Findings from these evaluations informed the formulation of personalized, stage-appropriate interventions.

**Figure 2:**
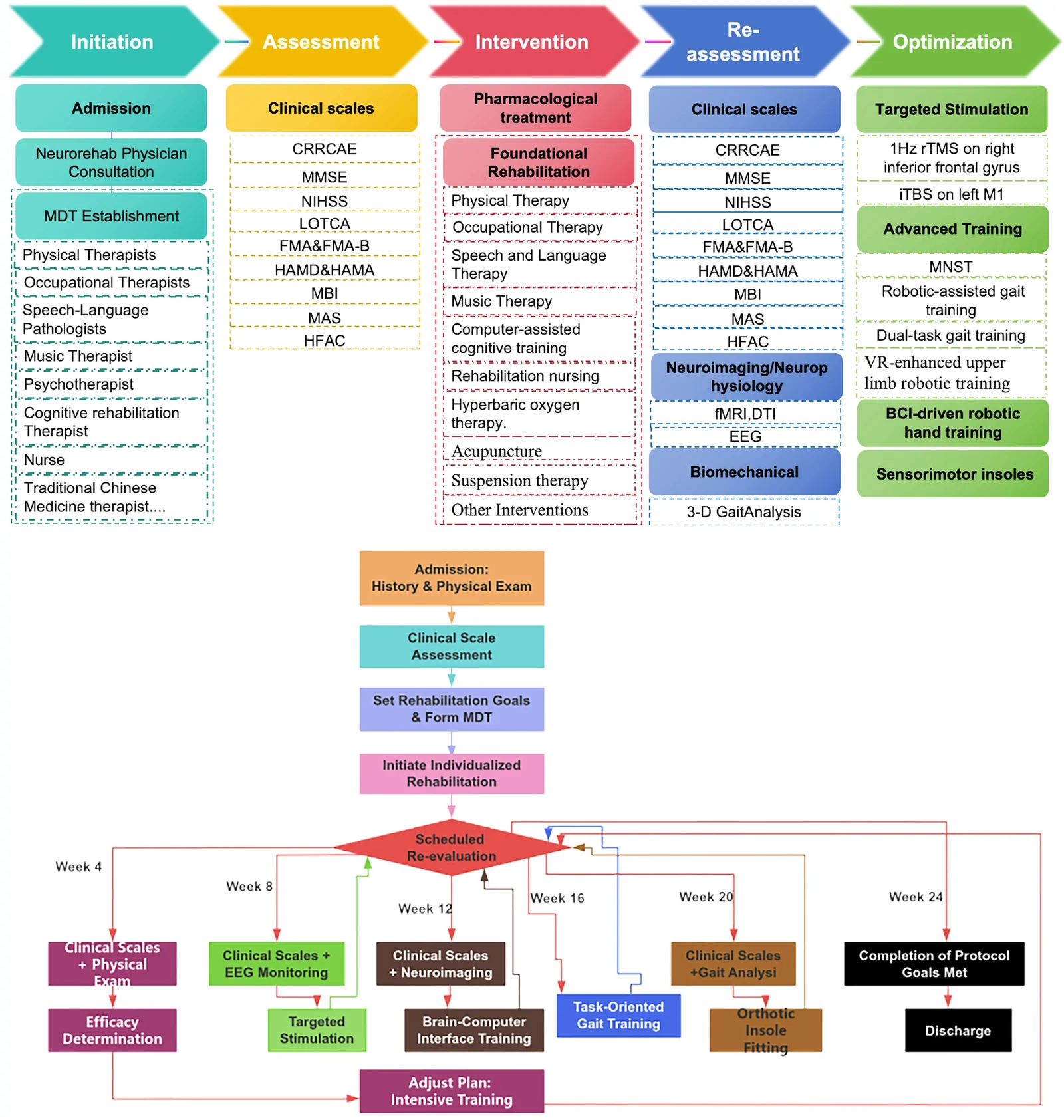
MDT-guided multimodal rehabilitation framework and timeline. Upper panel: The five phases of the MDT-guided rehabilitation framework – Initiation, Assessment, Intervention, Re-assessment, and Optimization - with their corresponding components. Clinical scales included CRRCAE, MMSE, NIHSS, LOTCA, FMA & FMA-B, HAMD & HAMA, MBI, MAS, and HFAC. Interventions included foundational rehabilitation, targeted stimulation (1 Hz rTMS and iTBS), advanced training (MNST, robotic-assisted gait training, VR-enhanced upper limb robotic training, BCI-driven robotic hand training), and sensorimotor insoles. Neuroimaging/neurophysiology included fMRI, DTI, and EEG; biomechanical assessment included 3-D gait analysis. Lower panel: The corresponding rehabilitation timeline. After admission and clinical scale assessment, rehabilitation goals were set and the MDT was formed. Individualized rehabilitation was initiated, with scheduled re-evaluation at Weeks 4, 8, 12, 16, 20, and 24. Targeted stimulation, BCI training, task-oriented gait training, and orthotic insole fitting were introduced at Weeks 8, 12, 16, and 20, respectively. At Week 24, the protocol goals were met and the patient was discharged. Abbreviations: BCI, brain-computer interface; CRRCAE, China Rehabilitation Research Center Aphasia Examination; DTI, diffusion tensor imaging; EEG, electroencephalography; FMA, Fugl-Meyer Assessment; FMA-B, Fugl-Meyer Assessment of Balance; fMRI, functional magnetic resonance imaging; HAMA, Hamilton Anxiety Scale; HAMD, Hamilton Depression Scale; HFAC, Holden Functional Ambulation Classification; iTBS, intermittent theta burst stimulation; LOTCA, Loewenstein Occupational Therapy Cognitive Assessment; MBI, modified Barthel Index; MDT, multidisciplinary team; MMSE, Mini-Mental State Examination; MNST, Mirror Neuron System Therapy; NIHSS, National Institutes of Health Stroke Scale; rTMS, repetitive transcranial magnetic stimulation; VR, virtual reality.

#### 2.2.1 MDT framework and assessment cycle

An MDT led by a neurorehabilitation physician was established, comprising physical therapists, occupational therapists, speech-language therapists, rehabilitation nurses, cognitive rehabilitation specialists, music therapists, psychologists, traditional Chinese medicine practitioners, and rehabilitation engineers. The team formulated a patient-centered, family-involved rehabilitation plan. A rehabilitation review meeting was held every four weeks to dynamically adjust the rehabilitation strategy based on the latest multimodal assessment results.

#### 2.2.2 Multimodal assessments

##### 2.2.2.1 Clinical scales

Chinese Rehabilitation Research Center Chinese Standard Aphasia Examination (CRRCAE), Mini-Mental Status Examination(MMSE),National Institutes of Health Stroke Scale(NIHSS),Loewenstein Occupational Therapy Cognitive Assessment(LOTCA),Fugl–Meyer Assessment(FMA),Fugl–Meyer Assessment of Balance Scale(FMA-B),Hamilton Anxiety Scale(HAMA),Hamilton Depression Scale(HAMD),Holden Functional Ambulation Classification(HFAC),MBI,and MAS.

##### 2.2.2.2 Neuroimaging

Resting-state functional MRI (rs-fMRI) and Diffusion Tensor Imaging (DTI) were performed on a Philips Achieva 3 T MRI scanner (Philips Medical Systems, Best, The Netherlands) using an 8-channel SENSE head coil. The rs-fMRI data were acquired using a gradient-echo echo-planar imaging (EPI) sequence with the following parameters: repetition time (TR) = 2,000 ms, echo time (TE) = 30 ms, flip angle = 75°, 37 axial slices, voxel size = 3 × 3 × 3 mm^3^, 200 volumes per run. DTI was acquired using a single-shot spin-echo EPI sequence with 32 gradient directions, TR = 9,900 ms, TE = 77 ms, flip angle = 90°, slice thickness = 2 mm, field of view = 224 × 224 mm^2^, and b-value = 1,000 s/mm^2^.

##### 2.2.2.3 Neurophysiology (EEG)

A 128-channel electroencephalography system (Neuracle, Beijing, China) was used for brain mapping and TMS target identification, with electrodes placed according to an extended 10–5 system (compatible with the international 10–20 layout) for high-density coverage. EEG signals were recorded at a sampling rate of 1,000 Hz with a bandpass filter of 0.5–100 Hz.

##### 2.2.2.4 Biomechanical

Dynamic three-dimensional gait analysis was performed using the Noraxon Ultium Motion system (Noraxon USA, Scottsdale, AZ, USA) with 16 inertial sensors, capturing spatiotemporal and kinetic parameters at a sampling rate of 400 Hz.

### 2.3 Therapeutic intervention

#### 2.3.1 Foundational rehabilitation

All guideline-based pharmacological agents (including antiplatelet, antihypertensive, lipid-lowering, and cognitive-enhancing) and conventional multidisciplinary therapies were delivered. These standard elements were systematically reframed within the context of filmmaking to enhance engagement and target high-level cognitive-communication functions:

**Physical Therapy (PT):** Targeting spasticity reduction, static/dynamic balance training, and gait re-education.
**Occupational Therapy (OT):** Emphasizing task-oriented activities of daily living (ADL) and functional hand skill retraining.
**Speech and Language Therapy (SLT):** Employing an integrated respiratory-phonatory-articulatory framework, augmented with semantic-lexical stimulation tasks (e.g., picture naming, auditory comprehension),mood matching, scene sequencing and Rosenbek eight-step protocol.
**Music Therapy:** Incorporating melodic intonation therapy (MIT), therapeutic singing, melodic-emotional processing to visual narrative choice, and structured musical memory exercises.
**Adjunctive Interventions:** Computer-assisted cognitive training, rehabilitation nursing, hyperbaric oxygen therapy, stationary cycling, multi-limb assisted movement, suspension therapy, acupuncture, and therapeutic massage.

All foundational sessions were conducted once daily, lasting 30 min, five days per week, over a 4-week block.

#### 2.3.2 Phase I (weeks 8∼12):targeted neuromodulation

##### 2.3.2.1 Targeted stimulation

Conventional therapy continued. Based on EEG showing right hemisphere hyper-excitability (Supplementary Figure S1), 1 Hz rTMS was applied to the right inferior frontal gyrus at 80% resting motor threshold, with 10 s of stimulation followed by 2 s of interstimulus interval, delivering 800 pulses per session (5 sessions/week for 4 weeks), to modulate interhemispheric inhibition for aphasia. Intermittent theta burst stimulation (iTBS) was applied to the left M1 using a standard paradigm: 2 s trains of three 50 Hz pulses repeated at 5 Hz, with 600 pulses per session at 80% active motor threshold (5 sessions/week for 4 weeks), for motor facilitation.

##### 2.3.2.2 Advanced training

The Mirror Neuron System Therapy (MNST v1.0, Suzhou, China), integrating immersive VR, action observation, motor imitation and auditory-linguistic stimuli, was introduced for synchronized multi-sensory input. This was complemented by task-specific advancements: in PT, dual-task and robotic-assisted gait training began; in OT, VR-enhanced upper limb robotic training (Wisebot® X7, Shenzhen, China) was introduced for hand function.

#### 2.3.3 Phase II (weeks 12∼16):BCI-VR integration

Following a plateau in upper limb FMA scores at Week 12, a hybrid BCI hand rehabilitation system, integrating motor imagery (MI) and steady-state visual evoked potential (SSVEP) technologies (9, 10)^,^ was introduced to provide additional intensive training targeting the persistent hand dysfunction. The training comprised two phases: (1) a calibration phase, in which the patient performed motor imagery of hand grasping (left/right random alternation) following visual cues without feedback (3 blocks of 3 min each, 20 trials per block, with 30 s rest between blocks); and (2) a closed-loop training phase, in which motor imagery of grasping triggered a soft robotic exoskeleton to assist the affected hand in completing the grasping movement, accompanied by visual feedback (3 blocks of 6 min each, 20 trials per block, 30 s rest between blocks). Each session lasted approximately 40 min, once daily, 5 days per week for 4 weeks. This BCI-driven robotic training was combined with task-oriented VR to enhance engagement and promote motor relearning.

#### 2.3.4 Phase III (weeks 20∼24):biomechanical optimization

Gait analysis revealed asymmetric compensatory patterns. The MDT referred the patient to orthotics, resulting in prescription of bilateral custom sensorimotor insoles (SMI) to improve alignment and weight-bearing. Concurrent physical therapy specifically targeted enhanced knee control and multi-joint coordination within functional balance training regimens.

#### 2.3.5 Phase Ⅳ:post-discharge follow-up

Following the completion of the 6-month inpatient rehabilitation program, the patient continued to receive outpatient follow-up care for an additional 2 months. During this period, the core therapeutic regimen was largely maintained, with fine-tuning based on periodic assessments to ensure the continuation and consolidation of rehabilitation gains.

(Detailed parameters for rTMS, iTBS, BCI are provided in Supplementary Table S1.).

### 2.4 Data analysis

#### 2.4.1 Diffusion tensor imaging (DTI)

DTI data were preprocessed and analyzed using FSL 6.0 (FMRIB Software Library, Oxford, UK) (11). Eddy current and head motion corrections were performed using FSL's eddy tool. Standard tract-based spatial statistics (TBSS) were applied to extract whole-brain fractional anisotropy (FA) values. Region-of-interest (ROI)-based fiber tractography was then performed using Diffusion Toolkit-TrackVis software (TrackVis, Boston, MA, USA) to visualize and quantify major white matter tracts,with FA threshold > 0.2 and angle threshold < 45°. The ROIs were placed over the corpus callosum and bilateral corticospinal tracts, anterior limbs of the internal capsules, and superior longitudinal fasciculi based on anatomical landmarks, and FA values were extracted for each identified tract.

#### 2.4.2 Resting-state fMRI (rs-fMRI)

rs-fMRI data were preprocessed using the RESTplus toolbox (version 1.2) (12) running in MATLAB (The MathWorks, Inc., Natick, MA, USA) within the SPM12 framework. The first five time points were discarded to allow for signal equilibrium. Preprocessing included slice-timing correction, realignment for head-motion correction using Friston's 24-parameter model, and co-registration of functional images to the individual structural image. No global signal regression was performed. Spatial smoothing was applied with a a 6-mm full-width at half-maximum (FWHM) Gaussian kernel. The preprocessed BOLD signals were transformed to the frequency domain via fast Fourier transform, and the amplitude of low-frequency fluctuations (ALFF) was calculated within the 0.01∼0.08 Hz frequency band. Regional homogeneity (ReHo) was calculated by measuring Kendall's coefficient of concordance between the time series of each voxel and its 26 nearest neighboring voxels. The automated anatomical labeling (AAL) atlas was used to parcellate the gray matter into 116 brain regions, and mean ALFF and ReHo values were extracted for each ROI.

#### 2.4.3 Electroencephalography (EEG) and event-related desynchronization (ERD)

EEG data recorded during BCI training sessions were analyzed offline using custom scripts in MATLAB.Data were re-referenced to the average reference, band-pass filtered at 0.5–100 Hz, and ocular artifacts were removed using independent component analysis (ICA). For each session, mu-band (8∼13 Hz) power was extracted from the sensorimotor region during motor imagery tasks, with a 500 ms pre-cue period used as baseline. A region of interest (ROI) was defined over the sensorimotor cortex, comprising electrodes FC3, FC1, FCz, FC2, FC4, C3, C1, Cz, C2, and C4. ERD was calculated as the percentage decrease in mu-band power during motor imagery relative to baseline. ERD values were extracted for each session across the 20 training sessions to examine the temporal trend in cortical excitability.

#### 2.4.4 Gait analysis

Three-dimensional gait data were processed using Noraxon Ultium Motion software (Noraxon USA, Scottsdale, AZ, USA) to extract spatiotemporal parameters (stride length, step length, step width, and walking speed), kinetic parameters (ground reaction force and plantar pressure distribution), and kinematic parameters (joint range of motion for the hip, knee, and ankle), as well as gait cycle phase parameters (stance and swing phase percentages).

## 3 Results

### 3.1 Multi-domain clinical functional recovery

Following the 6-month inpatient neurorehabilitation program and a 2-month follow-up, the patient’s functional status evolved from severe dependence to modified independence, with improvements observed across multiple domains (Table 1).

#### 3.1.1 Language

By the end of the intervention, the patient was able to use melodic intonation, read newspapers aloud, and comprehend his own scripts—a marked contrast to his admission status, when he was unable to perform any formal language task. Detailed CRRCAE subscale scores are presented in Supplementary Table S2 and Supplementary Video.

#### 3.1.2 Motor function

The patient transitioned from non-ambulatory status to independent ambulation indoors and outdoors. Right hand function remained limited (Brunnstrom stage III), restricting fine motor tasks.

#### 3.1.3 Cognition

Formal cognitive assessment (LOTCA) showed measurable gains, particularly in non-verbal domains (visuospatial organization and sequential thinking).

#### 3.1.4 Activities of daily living and participation

MBI score improved from 40/100 at admission to 91/100 at discharge. The patient regained the ability to perform basic self-care tasks and to engage in limited social interactions and personally meaningful activities (e.g., reading scripts, discussing films with family members).

### 3.2 Neuroimaging evidence of post-intervention neural changes

Figure 3 integrates multimodal neuroimaging findings documenting post-intervention neural plasticity:

**DTI (**Figures 3A–E**):** Post-intervention tractography showed increased fiber density. Quantitative analysis revealed increased FA in the corpus callosum (body/splenium) and the right corticospinal tract.
**Resting-state fMRI (**Figures 3F–G**):** Whole-brain voxel-wise analysis revealed elevated ALFF and ReHo in right-hemispheric regions, including the inferior frontal gyrus (pars triangularis), superior temporal gyrus, and middle temporal gyrus.

**Figure 3:**
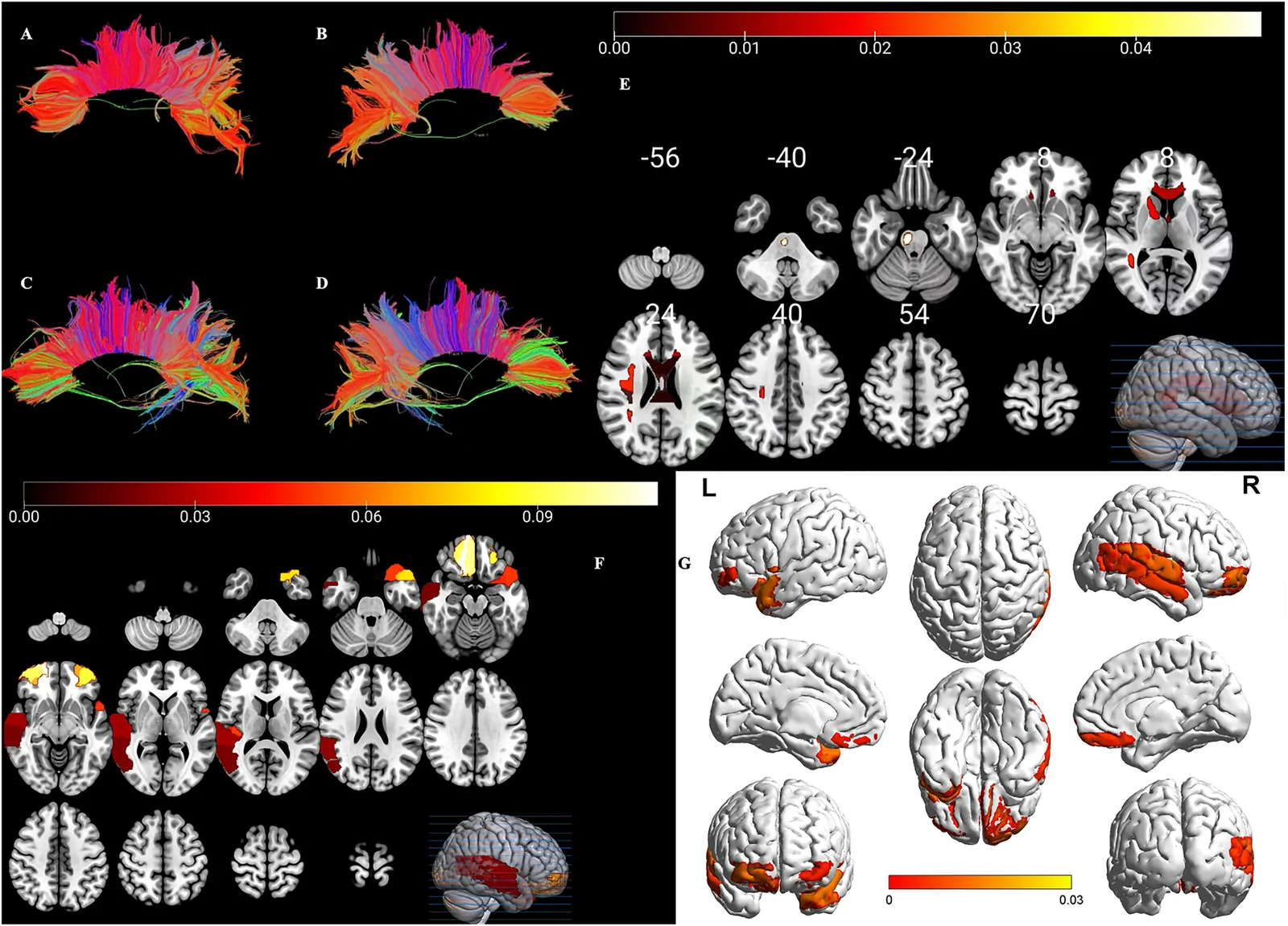
Multimodal neuroimaging evidence of neural changes. **(A–D)** Three-dimensional reconstruction of corpus callosum fiber tracking. A/B: Sagittal reconstruction of corpus callosum fibers before intervention (A: left lateral view; B: right lateral view). C/D: Sagittal reconstruction of corpus callosum fibers after intervention (C: left lateral view; D: right lateral view). **(E)** Regions showing increased fractional anisotropy (FA) after intervention (including the entire corpus callosum, right corticospinal tract, right anterior limb of internal capsule, and right superior longitudinal fasciculus). **(F,G)** Difference maps of resting-state functional indices (ALFF/ReHo) between pre- and post-intervention. F: Axial slices illustrating regions with increased ALFF. G: Brain surface maps depicting regions with increased ReHo. The color bar represents post-intervention minus pre-intervention difference values (*Δ* = Post−Pre). Warm colors indicate increase (*Δ* > 0), and cool colors indicate decrease (*Δ* < 0).

### 3.3 Electrophysiological changes during BCI training

Over the 20 BCI training sessions, mu-band (8–13 Hz) ERD over the sensorimotor cortex during motor imagery showed an overall downward trend, despite some session-to-session variability (Supplementary Figure S2). The topographic maps (Figures 4A,B) illustrate the spatial distribution of mu-band ERD before and after the 20-session BCI training, with enhanced desynchronization over the sensorimotor area following the intervention. For the left-hand motor imagery task, the majority of electrodes within the sensorimotor ROI (FC3, FC1, FCz, FC2, FC4, C3, C1, Cz, C2, C4) showed a decrease in ERD from Session 1 to Session 20, with the most pronounced changes observed over the central region (C3, C1, Cz, C2, C4). Quantitatively, the ROI-averaged ERD decreased from 0.35% in Session 1 to −0.79% in Session 20, with a negative linear trend (slope = −0.030). For the right-hand task, a similar downward trend was observed for most electrodes, with the ROI-averaged ERD decreasing from 0.36% in Session 1 to −0.80% in Session 20 (slope = −0.032).

**Figure 4:**
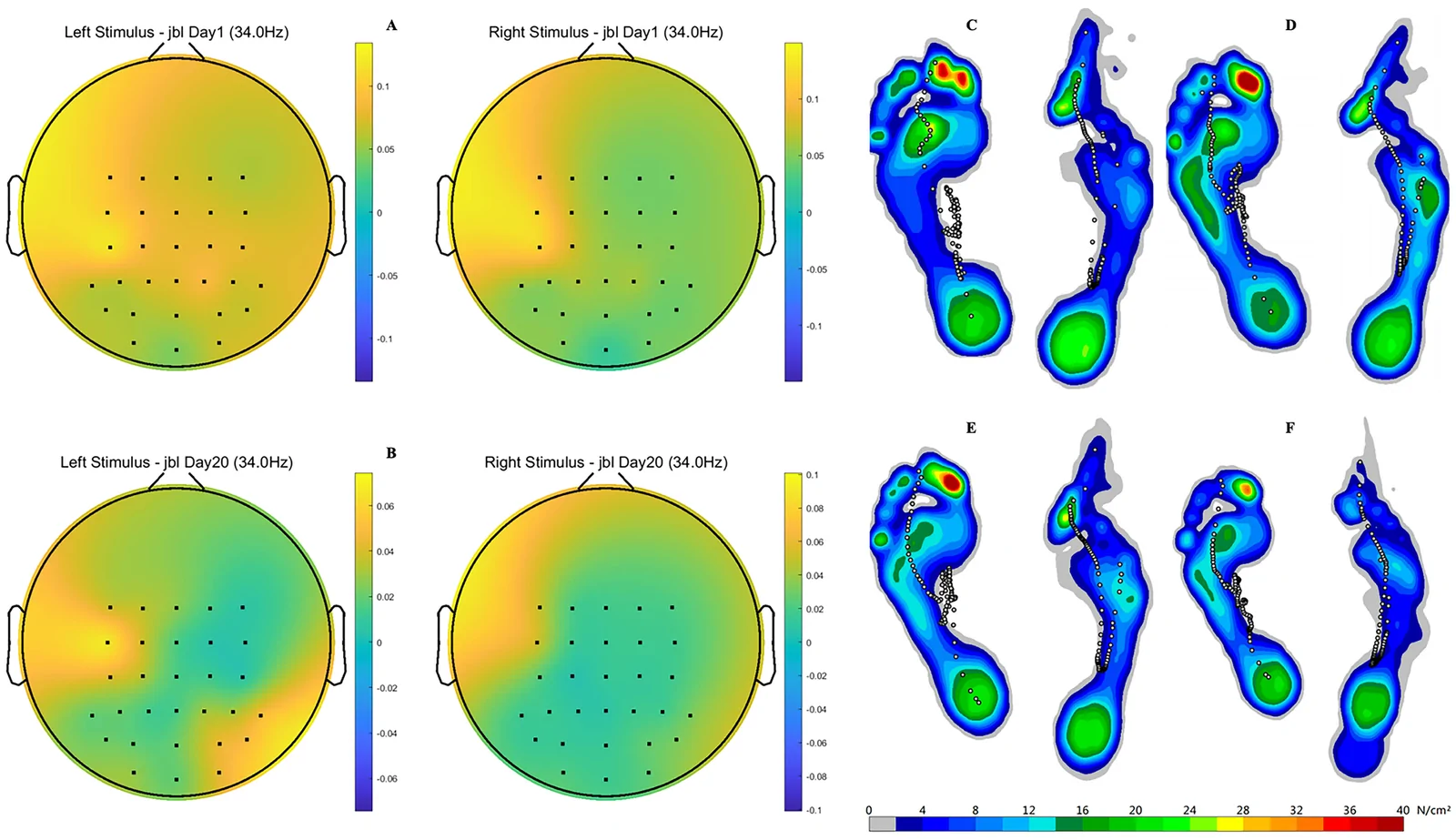
Neurophysiological and functional outcomes during BCI training and gait recovery. **(A,B)** Scalp topographic maps (top view) depicting the spatial distribution of mu-band (8∼13 Hz) event-related desynchronization (ERD) during motor imagery tasks before **(A)** and after **(B)** a 20-session closed-loop BCI hand function training regimen. Color bars represent ERD intensity, with cooler colors indicating stronger desynchronization. **(C–F)** Sequential plantar pressure distribution maps derived from 3D gait analysis, captured during the patient's transition from independent standing to independent walking at four key timepoints: **(C)** 12 weeks, **(D)** 16 weeks (following physical therapy regimen adjustment), **(E)** 20 weeks (on the day of custom foot insoles provision), and **(F)** 24 weeks (during continued insole use) of treatment.

### 3.4 Gait and biomechanical improvements

Longitudinal 3D gait analysis revealed improvements in stride length, walking speed, and plantar pressure symmetry by the end of the rehabilitation period (Figures 4C–F, Supplementary Table S3), with some fluctuations observed between assessments. A shift toward more symmetrical plantar pressure distribution was observed immediately after the introduction of custom SMIs at Week 20 and was maintained through the follow-up period.

## 4 Discussion

This case illustrates the application and potential value of a dynamic, MDT-guided closed-loop rehabilitation paradigm in a patient with severe, large-area stroke. The model's key feature lies in its systematic integration of serial, multimodal quantitative assessments to actively guide and adapt the staged implementation of targeted neuromodulation and adjunctive therapies (Figure 2), thereby transitioning from a fixed protocol to a responsive, precision-based approach.

### 4.1 Towards a mechanistic understanding: evidence for multi-level neural changes

The observed functional recovery coincided with objective neuroimaging and electrophysiological changes, offering preliminary observations that may reflect adaptive neural reorganization.

#### 4.1.1 Facilitating contralateral engagement and inter-hemispheric communication

The improvement in language and cognitive functions coincided with increased functional activity (ALFF/ReHo) in right frontal-temporal homologs and enhanced structural connectivity (FA) of the corpus callosum (Figure 3). These findings align with models of right-hemisphere compensatory involvement and suggest a potential role of strengthened inter-hemispheric connectivity in supporting functional recovery after major left hemispheric damage (13–15).

#### 4.1.2 Motor pathway integrity and cortical excitability

For recovery of the right (paretic) limb, the observed decrease in ERD over the sensorimotor cortex during BCI training was temporally associated with increased FA in the right corticospinal tract (Figures 4A,B). These changes occurred against the background of a comprehensive, multimodal rehabilitation program comprising both conventional therapies and advanced neuromodulatory techniques. Given the concurrent delivery of multiple interventions, the individual contribution of each modality cannot be isolated. However, the temporal associations observed in this case are consistent with the hypothesis that such integrated approaches may facilitate motor recovery through activity-dependent mechanisms (16–19).

These correlative observations are hypothesis-generating and do not establish causation; however, they are consistent with the possibility that right-hemisphere compensatory mechanisms and multimodal neuromodulation may contribute to recovery in this case.

### 4.2 The MDT as the engine for holistic and translational rehabilitation

Beyond neuromodulation, the MDT framework facilitated the translation of neurobiological changes into gains in activity and participation (5). Its strength lay in systematically addressing the functional consequences of injury across the ICF spectrum (2, 4). Crucially, the MDT operated on a cyclical assessment-and-reassessment model: at each review meeting, the team evaluated the patient's progress using the multimodal assessment battery and adapted the intervention strategy accordingly. This allowed the team to move beyond a fixed rehabilitation protocol and respond to the patient's evolving needs in a personalized, time-sensitive manner, which has been shown to positively influence psychosocial outcomes in stroke rehabilitation (6).

For example, when the patient showed limited gains after the initial 4-week block of conventional therapy — in language, upper limb, and gait domains — the MDT responded by introducing staged, targeted interventions (rTMS, BCI-based training, and custom insoles, respectively) rather than continuing a static routine. When 3D gait analysis revealed that gait dysfunction was not simply a unilateral problem but involved bilateral biomechanical compensatory patterns, the team referred the patient for custom SMIs (20) and adjusted physical therapy to focus on knee control and multi-joint coordination (21–23). This assessment-to-decision-to-adjustment process exemplifies the dynamic and personalized nature of the MDT approach.

### 4.3 Clinical implications, limitations and future directions

This case suggests a feasible template for personalizing complex stroke rehabilitation. The “assessment- to- decision” logic allows for the strategic deployment of resources by targeting specific, objectively identified recovery bottlenecks.

#### 4.3.1 Limitations

The primary limitations are inherent to the case report design, including the influence of individual variability and the inability to fully isolate treatment effects from natural recovery or from concurrent conventional therapies (2). Crucially, this patient's favorable prognostic profile — young age, absence of comorbidities, high functional reserve, and access to a prolonged, resource-intensive program in a specialized tertiary center — substantially limits the generalizability of our findings to older patients, those with multimorbidity, or healthcare systems with fewer resources. The resource intensity of the multimodal assessment battery also presents a challenge for broader implementation. Finally, because multiple interventions were delivered concurrently or in overlapping phases, no causal attribution can be made for any single modality; our descriptive associations should be interpreted as hypothesis-generating rather than confirmatory.

#### 4.3.2 Future directions

Future research should focus on validating this integrated model in larger cohort studies, identifying the most cost-effective and essential biomarker combinations, and exploring the use of advanced analytics to develop decision-support tools, thereby enhancing the scalability and precision of the closed-loop rehabilitation paradigm.

### 4.4 Summary

In summary, this report describes an MDT-coordinated, dynamically adjusted rehabilitation protocol incorporating targeted neuromodulation and holistic care that was temporally associated with notable functional recovery in a patient with large-area stroke. The accompanying objective neural measures provide preliminary, hypothesis-generating evidence for adaptive reorganization. While not without limitations, this closed-loop, precision framework highlights a promising direction for advancing the personalization and efficacy of neurorehabilitation in complex cases, meriting further investigation in controlled studies.

## Data Availability

The original contributions presented in the study are included in the article/Supplementary Material, further inquiries can be directed to the corresponding author.
