# Supplementary material for: Case Report: An MDT-guided multimodal protocol for large-area stroke plateau — integrating neuromodulation with multimodal assessment
Source: Front Rehabil Sci. 2026 Oct 9;7:1803123. doi: 10.3389/fresc.2026.1803123 (PMC13690806; doi:10.3389/fresc.2026.1803123)
Supplement: Supplementary file 1 [file Table1.docx]

| Parameter Category | rTMS Therapy (for Aphasia) | iTBS Therapy (for Motor Function) | BCI Training (for Upper Limb) |
| --- | --- | --- | --- |
| Treatment Target | Right inferior frontal gyrus, pars triangularis | Left primary motor cortex (M1) | Central-peripheral closed-loop system based on Motor Imagery |
| Method / Mode | 1 Hz repetitive Transcranial Magnetic Stimulation | Intermittent Theta Burst Stimulation (iTBS) | Motor Imagery + Steady-State Visually Evoked Potential (SSVEP) BCI |
| Stimulation / Training Parameters | • Intensity: 80% of Resting Motor Threshold (80% RMT) • Duration per Train: 10 seconds • Inter-train Interval: 2 seconds • Total Pulses: 800 per session | • Burst Pattern: 3 pulses at 50 Hz within a 2-second train • Intra-burst Frequency: 5 Hz • Pulses per Burst: 3 • Protocol: 10 bursts per train, repeated 20 times • Inter-train Interval: 8 seconds • Total Pulses: 600 per session | • Device: BCI soft robotic hand system • Session Duration: 40 minutes (including testing and training) |
| Treatment Protocol | • Frequency: Once daily • Sessions per Week: 5 • Total Course: 4 weeks (20 sessions total) | • Frequency: Once daily • Sessions per Week: 5 • Total Course: 4 weeks (20 sessions total) | • Frequency: Once daily • Sessions per Week: 5 • Total Course: 4 weeks (20 sessions total) |

**Table S2 Detailed parameters for rTMS, iTBS, BCI**

**Note:** This table summarizes the key parameters and protocols for the three neuromodulation interventions (rTMS, iTBS, and BCI training), facilitating comparison and reference. All treatments followed the same schedule of once daily, five times per week, for a total of 4 weeks.
