# Supplementary material for: Case Report: An MDT-guided multimodal protocol for large-area stroke plateau — integrating neuromodulation with multimodal assessment
Source: Front Rehabil Sci. 2026 Oct 9;7:1803123. doi: 10.3389/fresc.2026.1803123 (PMC13690806; doi:10.3389/fresc.2026.1803123)
Supplement: Supplementary file 2 [file Table2.docx]

**Table 2　 Pre- and Post-Treatment Variations in Key Indicators of the Chinese Rehabilitation Research Center Chinese Standard Aphasia Examination (CRRCAE)**

| Assessment Items | Nouns | | | Verbs | | | Sentences | | |
| --- | --- | --- | --- | --- | --- | --- | --- | --- | --- |
|  | At Admission | After 4 Weeks of Treatment | After 20 Weeks of Treatment | At Admission | After 4 Weeks of Treatment | After 20 Weeks of Treatment | At Admission | After 4 Weeks of Treatment | After 20 Weeks of Treatment |
| Auditory Comprehension(10 words) | 0 | 0 | 9 | 0 | 3 | 8 | 0 | 0 | 6 |
| Repetition(10words) | 0 | 3 | 10 | 0 | 3 | 9 | 0 | 0 | 6 |
| Speaking  (10words) | 0 | 0 | 4 | 0 | 0 | 4 | 0 | 0 | 2 |
| Oral Reading  (10words) | 0 | 0 | 3 | 0 | 0 | 5 | 0 | 0 | 2 |
| Reading  (10words) | 0 | 6 | 8 | 0 | 1 | 5 | 0 | 0 | 2 |
| Copying  (5words) | 0 | 0 | 3 | 0 | 0 | 3 | 0 | 0 | 1 |
