# Supplementary material for: Case Report: An MDT-guided multimodal protocol for large-area stroke plateau — integrating neuromodulation with multimodal assessment
Source: Front Rehabil Sci. 2026 Oct 9;7:1803123. doi: 10.3389/fresc.2026.1803123 (PMC13690806; doi:10.3389/fresc.2026.1803123)
Supplement: Supplementary file 3 [file Table3.docx]

| **Table 3. Changes in Gait Analysis Indicators During Rehabilitation Training** | | | | |
| --- | --- | --- | --- | --- |
| Indicator | After 12 Weeks of Treatment | After 16 Weeks of Treatment | After 20 Weeks of Treatment | At Discharge |
| Stance Phase Percentage % (Left/Right) | 92.4/85.5 | 91.7/76.4 | 91.9/84.6 | 88.1/78.4 |
| Stride Length (cm) | 44±3 | 55±3 | 54±4 | 49±3 |
| Step Length (Left, cm) | 13±3 | 23±3 | 25±1 | 24±1 |
| Step Length (Right, cm) | 30±0 | 32±4 | 30±4 | 25±2 |
| Step Width (cm) | 21±0 | 17±1 | 18±1 | 15±1 |
| Double Support Phase Percentage % | 74.0±0.8 | 64.4±5.6 | 73.8±4.5 | 68.3±5.5 |
| Swing Phase Percentage % (Left/Right) | 7.6/14.5 | 8.3/23.6 | 8.1/15.4 | 11.9/21.6 |
| Walking Speed (km/h) | 0.4±0.0 | 0.5±0.0 | 0.4±0.0 | 0.5±0.0 |
| Ground Reaction Force (Left/Right, N) | 57.8/1.5 | 22.4/3.76 | 85.6/4.08 | 19.7/2.34 |
| Hip Flexion Range of Motion (Left) | -5.49 | -6 | -4.68 | -6.25 |
| Hip Flexion Range of Motion (Right) | -8.04 | -3.57 | 0.85 | -7.54 |
| Knee Flexion Range of Motion (Left) | -0.508 | 3.58 | 4.83 | 3.47 |
| Knee Flexion Range of Motion (Right) | -6.81 | -2.52 | 24.1 | -5.68 |
| Ankle Dorsiflexion Range of Motion (Left) | -20.2 | -10.8 | -14.1 | -10.4 |
| Ankle Dorsiflexion Range of Motion (Right) | -14.1 | -9.94 | -6.06 | -2.5 |
| Ankle Inversion (Left) | -5.48 | -9.75 | -8.27 | 0.0428 |
| Ankle Inversion (Right) | 1.6 | 5.76 | 12.1 | -2.19 |
| Heel Pressure (Left/Right, N) | 436.8/519.8 | 343.6/484.1 | 376.2/468.4 | 370.9/516.4 |
| Midfoot Pressure (Left/Right, N) | 153.2/208.1 | 319.1/301.9 | 225.2/284.6 | 176.8/159.0 |
| Forefoot Pressure (Left/Right, N) | 449.6/159.9 | 579.4/132.2 | 502.2/153.7 | 460.2/255.9 |
